# Physical activity, social support, and working memory in older adults: evidence from a Chinese sample

**DOI:** 10.3389/fpsyg.2026.1771223

**Published:** 2026-04-29

**Authors:** Jianing Lyu, Ling Zhang, Mingsheng Xiong

**Affiliations:** School of Psychology, Wuhan Sports University, Wuhan, China

**Keywords:** older adults, moderation effect, n-back task, physical activity, social support, working memory

## Abstract

This study examined the associations between physical activity (PA), social support and working memory performance in older adults. With a particular focus on the potential moderating role of social support. Using a cross-sectional design, we recruited 123 community-dwelling older adults who completed assessments of PA (MET-min/week), social support (SSRS total score), and working memory performance across 0-back, 1-back, and 2-back conditions. After standardizing PA and SSRS, an interaction term was constructed. Hierarchical regression analyses were conducted after standardizing variables, with age, sex, and education controlled. The results indicated that both PA and social support were positively associated with 2-back accuracy, while no significant associations were observed for 0-back or 1-back performance. A significant moderating effect of social support was observed, such that the association between PA and working memory was stronger at higher levels of social support. These findings suggest that higher levels of physical activity and social support are associated with better high-load working memory performance, and that social support may enhance the cognitive benefits of physical activity in older adults.

## 1 Introduction

With the rapid acceleration of global population aging, challenges related to elderly care, healthcare systems, and social security have become increasingly prominent (United Nations Department of Economic and Social Affairs, 2023). According to projections by the United Nations, the global population aged 65 years and older is expected to approach 1.6 billion by 2050, accounting for more than 16% of the total population. Changes in population structure have also made the protection of cognitive function in older adults a central concern of societal and scientific attention (Wu, 2012). Since the promulgation of the National Fitness Plan (2014–2020) by the State Council of China in 2014, the national fitness strategy—emphasizing “participation by all and lifelong benefits” has been vigorously promoted. In China, the National Fitness Program (State Council of the People’s Republic of China, 2016) has identified older adults as a key target group. This policy framework has provided institutional support for enhancing both physical and psychological health among older adults. However, in practice, challenges remain, including insufficient motivation for physical activity and relatively weak social support networks (Erickson et al., 2011).

One of the most prominent health challenges in later life is the decline in cognitive function. Previous research has shown that age-related cognitive decline is not limited to reductions in memory and attentional capacity, but also broadly involves core cognitive components such as executive function, information processing speed, and working memory (Stillman et al., 2020; Li et al., 2022). These declines substantially undermine older adults’ independence in daily living and levels of social participation, while simultaneously increasing the risk of mild cognitive impairment and neurodegenerative disorders such as Alzheimer’s disease (Harada et al., 2013). Consequently, identifying effective intervention pathways to promote cognitive health is of critical importance for achieving healthy aging. In this context, examining the interactive effects of physical activity and social support holds considerable practical relevance and theoretical significance (Petersen et al., 1999).

Among the various dimensions of cognitive function, working memory (WM) is considered one of the most central components and one of the most susceptible to age-related decline (Salthouse, 2010). Working memory refers to the capacity to temporarily maintain and manipulate information over short periods of time, serving as a fundamental cognitive mechanism underlying learning, reasoning, and executive control (Salthouse, 2019). According to Baddeley’s multicomponent model, working memory comprises several interacting subsystems, including the phonological loop, the visuospatial sketchpad, and the central executive, which together support the short-term storage and processing of information (Baddeley, 2012). With advancing age, working memory capacity typically decreases, processing speed slows, and resistance to interference weakens. Consequently, working memory represents both a core indicator of cognitive aging and a key target for cognitive intervention research (Baddeley, 2003). Consistent evidence further demonstrates that working memory performance in the n-back task declines as task load increases, with older adults showing particular sensitivity to higher levels of cognitive demand (Owen et al., 2005).

Regular physical activity (PA), particularly moderate-to-vigorous aerobic exercise, has been consistently associated with executive function and working memory in later life. Seminal meta-analytic evidence indicates that fitness training exerts robust and selective benefits on cognitive functioning in older adults, with gains predominantly observed in higher-order processes such as executive control, including components of working memory (Colcombe and Kramer, 2003). Randomized controlled trials have further demonstrated that aerobic exercise training can improve memory performance (Hillman et al., 2008). Subsequent reviews and recent intervention studies have similarly shown that aerobic exercise enhances global cognitive functioning as well as working memory and updating processes in older adults, although the magnitude of these effects may vary as a function of exercise prescription and individual differences (Voss et al., 2011). Evidence from large-scale studies in China further supports a positive association between physical activity and cognitive functioning among middle-aged and older adults (Chen et al., 2025; Zhao et al., 2022). Within the Chinese cultural context, moderate-intensity activities such as Tai Chi are widely practiced among older adults. Systematic reviews and integrative analyses indicate that Tai Chi interventions lasting at least 10 weeks, performed three or more times per week with sessions of at least 30 min, can significantly improve executive function, verbal fluency, and performance related to working memory in older populations (Voss et al., 2013).

Social support is widely regarded as a key psychosocial resource that associated with cognitive functioning and health-related behaviors through mechanisms such as stress buffering (Cohen and Wills, 1985). Longitudinal evidence indicates that higher levels of social connectedness and support are associated with slower rates of cognitive decline in older adults (Seeman et al., 2001). At the physiological level, social support has been linked to more favorable cardiovascular, neuroendocrine, and immune functioning, highlighting its role in shaping health outcomes through multiple biological pathways (Uchino, 2006). At a broader population-health level, a large-scale meta-analysis involving more than 300,000 participants demonstrated a significant association between the strength of social relationships and survival rates, underscoring social support as a critical determinant of health (Holt-Lunstad et al., 2010). In addition, social participation—an important manifestation of social support—has been shown to exert significant positive effects on both cognitive and psychological health among older adults (Xue et al., 2018), suggesting that social networks and active engagement constitute potential psychosocial mechanisms for maintaining cognitive functioning in later life. Recent research has increasingly highlighted the role of both physical activity and social support in cognitive aging. Longitudinal evidence suggests that physical activity is associated with better cognitive functioning over time, particularly in the context of social relationships (Cohn-Schwartz et al., 2020). Systematic reviews have further shown that social support is positively associated with cognitive functioning across multiple domains (Costa-Cordella et al., 2021). Both theoretical frameworks and empirical findings suggest that social support has been suggested to be associated with factors such as greater behavioral adherence and positive affect, which may, in turn, relate to cognitive functioning. Accordingly, examining the moderating role of social support in the pathway from physical activity to working memory is of substantial theoretical and practical significance, particularly for community-based health promotion and individualized intervention strategies (Holt-Lunstad et al., 2015).

Longitudinal studies have demonstrated that older adults with higher levels of social support experience a slower rate of cognitive decline, and this association remains significant even after controlling for education, health status, and related covariates (Seeman et al., 2001). The underlying mechanisms of this effect appear to operate primarily through two pathways. First, social support can buffer stress, thereby reducing negative affect and physiological load (Holt-Lunstad et al., 2015). Second, by activating psychosocial resources, social support enhances self-efficacy and social participation, which in turn indirectly promotes cognitive functioning. Evidence from studies conducted in China further suggests that social support not only directly predicts cognitive performance in older adults, but also indirectly improves cognition by alleviating depressive symptoms, enhancing sleep quality, and increasing life satisfaction. In this context, intergenerational support and emotional companionship have been identified as particularly important protective factors for psychological well-being and cognitive health in later life. In contrast, physical activity primarily promotes cognitive functioning through improvements in brain structure and neural plasticity, whereas social support exerts its effects mainly via psychosocial mechanisms that enhance motivation and emotion regulation (Erickson et al., 2011; Stillman et al., 2020). Higher levels of social support may therefore facilitate greater adherence to physical activity and more positive exercise experiences, thereby amplifying the beneficial effects of physical activity on executive function and working memory (Smith et al., 2010). Elucidating the moderating role of social support in the relationship between physical activity and working memory not only helps to explain individual differences in cognitive aging, but also provides a theoretical foundation for the development of culturally tailored interventions aimed at promoting cognitive health in older adults.

Although prior research has separately examined the associations among physical activity, social support, and cognitive functioning, the potential interactive mechanisms among these variables remain insufficiently explored. In particular, among older adults, physical activity and social support may jointly associated with cognitive functioning through synergistic effects; however, this perspective has received limited systematic and mechanistic empirical investigation. Existing studies have predominantly focused on the independent effects of physical activity or social support on cognitive outcomes, with relatively few examining their interaction. Moreover, prior research has typically used global cognitive functioning or executive function as outcome variables, with comparatively limited attention to working memory as a core cognitive component—especially under varying levels of cognitive load. In addition, empirical evidence based on community-dwelling older adults in China remains relatively scarce. Given these gaps, it is necessary to further investigate how physical activity and social support jointly associated with working memory performance in older adults.

Taken together, existing evidence suggests that physical activity, as a critical lifestyle factor for promoting cognitive health in later life, not only exerts its effects through improvements in brain structure and neural plasticity (Erickson et al., 2011), but may also interact with psychosocial resources such as social support to associated with cognitive functioning. Empirical findings from studies conducted in China have further corroborated this pattern (Voss et al., 2011). For example, Bai et al. (2011) reported that regular physical activity significantly enhances executive function and memory performance among older adults. Using data from the China Health and Retirement Longitudinal Study (CHARLS), Xue et al. (2018) demonstrated that social participation and social support are associated with a slower rate of cognitive decline in middle-aged and older adults. More recently, provided additional evidence indicating that social support, physical activity, and educational attainment are important predictors of cognitive health in aging populations (Wang et al., 2023).

From a theoretical perspective, the present study is informed by two conceptually distinct yet complementary frameworks. Cognitive reserve theory posits that sustained engagement in physical activity contributes to neural plasticity and the accumulation of cognitive reserve, thereby supporting cognitive functioning in later life (Stern, 2009). In contrast, the social support buffering model emphasizes that social support operates primarily through psychosocial pathways, such as reducing stress, enhancing emotional well-being, and promoting behavioral adherence (Cohen and Wills, 1985).

Importantly. Importantly, the theoretical distinction between these mechanisms provides a basis for specifying social support as a moderator rather than a mediator. A moderator is defined as a variable that influences the strength or direction of the relationship between an independent variable and an outcome. While mediation would imply that physical activity influences cognitive outcomes through changes in social support, the present theoretical framework does not assume such a directional pathway. Instead, social support is conceptualized as a relatively stable contextual resource that shapes how effectively individuals engage in and benefit from physical activity. Similarly, treating social support as a covariate or correlated factor would fail to capture its role in modifying the strength of the association between physical activity and cognitive functioning. Therefore, consistent with the stress-buffering model, social support is more appropriately specified as a moderator that amplifies or attenuates the cognitive benefits of physical activity.that may jointly be associated with working memory performance. This distinction provides the theoretical basis for examining the moderating role of social support in the association between physical activity and working memory.

Accordingly, the present study contributes to the literature in three key ways. First, it moves beyond examining independent effects to investigate interactive mechanisms by testing the moderating role of social support in the relationship between physical activity and working memory, thereby elucidating their joint associated with on cognitive functioning. Second, it refines the assessment of cognitive processes by incorporating varying levels of cognitive load, with a particular focus on high-load (2-back) working memory performance. Third, by focusing on community-dwelling older adults in China, this study provides contextually grounded evidence within a specific sociocultural and policy framework, thereby extending the external validity and practical relevance of existing research.

Based on the above theoretical and empirical foundations, the present study focuses on community-dwelling older adults in China to examine the effects of physical activity and social support on working memory, as well as their interaction. The following hypotheses are proposed:

*H1*: Physical activity is positively associated with working memory performance.

*H2*: Social support is positively associated with working memory performance.

*H3*: The positive association between physical activity and working memory performance is stronger at higher levels of social support.

## 2 Materials and methods

### 2.1 Participants

#### 2.1.1 Sample recruitment

Participants were recruited in two stages through community-based sampling from neighborhood committees, universities for older adults, and public fitness centers. The initial sample was followed by supplementary recruitment aimed at improving the representation of older adults with low levels of physical activity. From community neighborhood committees, universities for older adults, and public fitness centers. A total of 147 healthy older adults initially participated in the study. Following data quality control procedures, 24 participants were excluded due to incomplete questionnaires, n-back task response times shorter than 200 ms or longer than 3,000 ms, or task accuracy values exceeding ±3 standard deviations from the mean. The final analytic sample therefore consisted of 123 participants aged 59–80 years (*M* = 67.54, SD = 5.147). This sample size is considered adequate for detecting moderation effects and is comparable to those reported in previous studies employing similar analytical approaches (Hayes, 2022), as shown in Table 1. Participants were primarily recruited from residential communities affiliated with local universities and surrounding neighborhoods. Overall, the sample demonstrated a relatively high level of educational attainment, with 36 participants (29.3%) having completed junior high school or below, 44 participants (35.8%) having completed senior high school, and 43 participants (35.0%) holding a bachelor’s degree or above. This educational background ensured adequate comprehension of the questionnaire items and proper performance of the experimental tasks. Group comparison analyses indicated no significant differences in age (*F* = 0.55, *p* = 0.581), sex (*χ^2^* = 0.20, *p* = 0.905), and educational level (*F* = 0.55, *p* = 0.576) among participants categorized into low, moderate, and high physical activity groups, suggesting that the sample was comparable across physical activity levels.

**Table 1:** Demographic characteristics of participants (*N* = 123).

Group	*n*	Male (%)	Female (%)	Age	Years of education
Low	25	56.0	44.0	66.80 ± 5.34	8.21 ± 3.41
Moderate	57	47.4	52.6	68.09 ± 4.37	8.67 ± 3.11
High	41	46.3	53.7	68.95 ± 4.12	8.85 ± 2.98

#### 2.1.2 Inclusion and exclusion criteria

To ensure the scientific rigor, representativeness of the sample, and validity of the study findings, inclusion and exclusion criteria were established in accordance with standard practices in research on cognitive aging.

Inclusion criteria were as follows: (1) age ≥ 59 years; (2) ability to independently complete questionnaire assessments and computerized experimental tasks; (3) normal or corrected-to-normal vision and hearing sufficient to meet the requirements of task performance and stimulus presentation.

Exclusion criteria included: (1) presence of severe neurological disorders, such as Alzheimer’s disease or Parkinson’s disease; (2) severe visual or auditory impairments that could not be adequately corrected and would prevent accurate task completion; (3) use of medications that may affect central nervous system functioning (e.g., sedatives, antidepressants, or anxiolytics) within the past 3 months; (4) participation in cognitive training programs or other similar psychological experiments within the past six months.

#### 2.1.3 Informed consent

This study was conducted in strict accordance with the ethical principles outlined in the Declaration of Helsinki (World Medical Association, 2013). Prior to participation, all participants received a detailed explanation of the study and voluntarily provided written informed consent, indicating that they were fully informed of the study’s purpose, procedures, and their rights as participants. Participants were informed that they could withdraw from the study at any time without penalty or adverse consequences. All data were collected solely for research purposes and were processed anonymously to ensure confidentiality.

### 2.2 Measures

#### 2.2.1 Assessment of physical activity

Physical activity (PA) was assessed using the International Physical Activity Questionnaire–Short Form (IPAQ-SF) (IPAQ Research Committee, 2005). The IPAQ-SF, developed by Craig and colleagues, is designed to evaluate the intensity and duration of physical activity performed over the past seven days (Craig et al., 2003). The questionnaire has demonstrated good test–retest reliability (*r* = 0.76–0.94) and acceptable criterion validity across samples from 12 countries (Macfarlane et al., 2007). The Chinese version of the IPAQ-SF has also been validated in older adult populations in China, showing satisfactory internal consistency and validity (Deng et al., 2008).

The IPAQ-SF consists of seven items assessing three categories of physical activity: vigorous-intensity activities (e.g., running, fast walking), moderate-intensity activities (e.g., Tai Chi, household chores), and walking. Sitting time is recorded for descriptive comparison only and is not included in the calculation of total physical activity. The metabolic equivalent of task (MET) values assigned to each activity category are presented in Table 2. Energy expenditure for each activity was calculated using the following formula: MET-min/week = MET value × number of days per week × duration per day (minutes).

**Table 2:** Physical activity measurement indicators for older adults based on the IPAQ.

Physical activity domain	Activity type	Intensity level	MET Value
Work	Walking	Walking	3.3
	Moderate-intensity activity	Moderate	4
	Vigorous-intensity activity	Vigorous	8
Transportation	Walking	Walking	3.3
	Cycling	Moderate	6
Domestic and Gardening Activities	Moderate-intensity indoor housework	Moderate	3
	Moderate-intensity outdoor housework	Moderate	4
	Vigorous-intensity outdoor housework	Moderate	5
Leisure-Time Activities	Walking	Walking	3.3
	Moderate-intensity activity	Moderate	4
	Vigorous-intensity activity	Vigorous	8

MET, metabolic equivalent of task. MET values are assigned according to the IPAQ scoring protocol.

Total physical activity was computed as the sum of MET-min/week across the three activity categories, excluding sitting time. According to the official IPAQ classification criteria (Craig et al., 2003), participants were categorized into high (≥1,500 MET-min/week from vigorous activity or ≥3,000 MET-min/week from any combination), moderate (≥600 MET-min/week), and low (<600 MET-min/week) physical activity groups (see Table 3). This classification approach has been validated in older adult populations in China (Macfarlane et al., 2007; Deng et al., 2008), demonstrating satisfactory reliability and validity. All questionnaire data were independently entered and cross-checked by two trained researchers. The discrepancy rate was below 1%, ensuring the reliability and accuracy of the data.

**Table 3:** Criteria for classifying individual physical activity levels.

Category	Criteria
High	Meets either of the following two criteria:
	(1) Vigorous-intensity physical activity on ≥3 days, with a total physical activity level of ≥1,500 MET-min/week
	(2) Physical activity of any combination of intensities on ≥7 days, with a total physical activity level of ≥3,000 MET-min/week
Moderate	Meets any one of the following three criteria:
	(1) Vigorous-intensity physical activity for ≥20 min/day on ≥3 days
	(2) Moderate-intensity physical activity and/or walking for ≥30 min/day on ≥5 days
	(3) Physical activity of any combination of intensities on ≥5 days, with a total physical activity level of ≥600 MET-min/week
Low	Meets either of the following two criteria:
	(1) No physical activity reported
	(2) Some physical activity reported, but not sufficient to meet the criteria for the moderate or high physical activity categories

Classification criteria are based on the official IPAQ scoring guidelines.

#### 2.2.2 Assessment of working memory

Working memory processing and updating were assessed using the classic N-back paradigm (Jaeggi et al., 2010). The N-back paradigm is widely used to assess working memory under varying cognitive loads. In older adults, 0-back, 1-back, and 2-back conditions are commonly adopted to represent increasing task demands while maintaining feasibility (Owen et al., 2005; Jaeggi et al., 2010). The task was programmed and administered using E-Prime 3.0 software, with randomly presented digits (0–9) as stimuli. The experiment included three load conditions: 0-back, 1-back, and 2-back. In the 0-back condition, Participants were required to determine whether the current digit was “0.” In the 1-back and 2-back conditions, participants judged whether the current digit matched the digit presented one or two trials earlier, respectively (Kirchner, 1958).

Responses were recorded via a two-key response system (“*n*” = match/same; “*v*” = non-match/different) (Owen et al., 2005). Accuracy in the 2-back condition was selected as the primary outcome measure of working memory performance. Each condition comprised 60 trials, including 20 target trials and 40 non-target trials, with digits (0–9) presented in a randomized order. Target stimuli accounted for 33% of trials, yielding a target-to-non-target ratio of 1:2. Each stimulus was displayed for 700 ms, followed by an interstimulus interval (ISI) of 2000 ms. Prior to the formal experiment, participants completed six practice trials to familiarize themselves with the task procedure. Additional practice trials were provided as needed until an accuracy rate of at least 80% was achieved or until the practice limit was reached. The order of the three load conditions was counterbalanced using a Latin square design (Redick and Lindsey, 2013). The entire task lasted approximately 12–15 min. Working memory performance was primarily indexed by accuracy (ACC), calculated as the proportion of correct responses across trials. Reaction time (RT) was recorded as a secondary measure and was calculated based on correct responses only. For each participant, mean RTs were computed across correct trials within each condition. Trials with RTs shorter than 200 ms or longer than 3,000 ms were excluded from analysis to minimize the influence of anticipatory or delayed responses. In addition, participants with accuracy or RT values exceeding ±3 standard deviations from the sample mean were excluded from further analysis.

#### 2.2.3 Assessment of social support

Social support was assessed using the Social Support Rating Scale (SSRS) developed by Xiao (1994). The SSRS comprises 10 items and consists of three dimensions: subjective support (4 items), reflecting individuals’ perceived emotional support; objective support (3 items), assessing tangible or instrumental support actually received; and support utilization (3 items), indicating the extent to which individuals seek and make use of social support resources when facing difficulties. In the present sample, the SSRS demonstrated good internal consistency reliability.

Scoring procedures were as follows: Items Q1–Q4 and Q8–Q10 were rated on a 4-point Likert scale (1–4). Item Q5 includes four subcomponents (spouse, parents, children/siblings, and other relatives or friends), each rated from 1 (no support) to 4 (full support), with the sum representing the item score. Items Q6 and Q7 are multiple-response items and were scored based on the number of sources endorsed (with *n* sources scored as *n* points), which were subsequently converted to a 4-point scale. The total SSRS score ranges from 12 to 66, with higher scores indicating higher levels of perceived or received social support. According to the original classification criteria (Xiao, 1994), total scores of ≥40 indicate high social support, scores of 20–39 indicate moderate social support, and scores of <20 indicate low social support.

### 2.3 Procedure and data analysis

Data were collected using a one-on-one testing procedure. All experimenters received standardized training and followed a uniform protocol when explaining the tasks, ensuring that participants completed the assessments independently after fully understanding the instructions. After providing written informed consent, participants completed the International Physical Activity Questionnaire–Short Form (IPAQ-SF; Craig et al., 2003) and the Social Support Rating Scale (SSRS; Xiao, 1994), followed by the n-back working memory task, which included the 0-back, 1-back, and 2-back conditions. The entire experimental session lasted approximately 20 min on average. Data entry and quality control were independently conducted by two researchers, with a discrepancy rate below 1%. Participants with incomplete questionnaires or abnormal response times (response times < 200 ms or > 3,000 ms, or values exceeding ±3 standard deviations from the mean) were excluded. The final analytic sample consisted of 123 valid participants. The study employed a 3 (physical activity level: high, moderate, low; between-subjects factor) × 3 (n-back task difficulty: 0-back, 1-back, 2-back; within-subjects factor) mixed experimental design. Physical activity level served as the between-subjects independent variable, task difficulty as the within-subjects independent variable, and social support (total SSRS score) as the moderating variable. All tasks were conducted in a quiet laboratory with appropriate lighting, and experimental conditions were kept consistent across participants to ensure comparability of testing conditions.

Statistical analyses were performed using SPSS 26.0. Moderation analyses were conducted using the PROCESS macro (Model 1, version 4.1; Hayes, 2022), and figures were generated using Origin 2023 for data visualization, Ethical approval and research procedures complied with the Declaration of Helsinki (World Medical Association, 2013). Reliability analyses indicated that all measurement instruments demonstrated satisfactory reliability and structural validity. To provide a comprehensive understanding of the data, multiple analytical approaches were employed with distinct purposes. Repeated-measures ANOVA was used to examine differences in working memory performance across task conditions (0-back, 1-back, and 2-back), serving a descriptive role. Pearson correlation analyses were conducted to explore bivariate associations among the key variables.

The primary hypotheses were tested using regression analyses. The primary hypotheses were tested using hierarchical regression analyses. Continuous variables were standardized prior to analysis, and an interaction term (physical activity MET × social support) was computed. Age, sex, and education were included as covariates. Moderation effects were further examined using the PROCESS macro (Model 1; Hayes, 2022). A bootstrap procedure with 5,000 resamples was used to estimate 95% confidence intervals, providing a robust test of interaction effects without relying on normality assumptions. Multicollinearity diagnostics indicated that all variance inflation factor (VIF) values were below 1.3. Group-based analyses (low, moderate, high physical activity) were conducted for descriptive purposes only. To address potential bias associated with unequal subgroup sizes, all primary analyses were based on continuous physical activity (MET) measures.

## 3 Results

### 3.1 Common method bias

As all questionnaire data in the present study were collected via self-report paper-and-pencil measures, the potential risk of common method bias (CMB) was examined using Harman’s single-factor test. An unrotated exploratory factor analysis extracted four factors with eigenvalues greater than 1, and the first factor accounted for 38.5% of the total variance. This value was below the commonly accepted threshold of 40%, indicating that no serious common method bias was present and that the discriminant validity among the study variables was acceptable.

### 3.2 Descriptive statistics and reliability

To ensure the reliability of the measurement instruments and experimental tasks, descriptive statistics and reliability analyses were conducted for the main variables prior to the formal analyses. The distributions of all variables met the assumptions of normality, with absolute values of skewness and kurtosis below 1, indicating that the data were suitable for parametric analyses. A total of 123 healthy older adults were included in the final sample (see Table 1).

With respect to social support, the mean total score on the Social Support Rating Scale (SSRS) was *M* = 36.28 (*SD* = 4.18). Based on the 10-item scale, the SSRS demonstrated excellent internal consistency reliability in the present sample (Cronbach’s *α* = 0.93), which is consistent with reliability estimates reported in previous studies. Reliability coefficients for the three subscales were also satisfactory, with *α* = 0.85 for subjective support, *α* = 0.79 for objective support, and *α* = 0.83 for support utilization.indicating satisfactory internal consistency and structural validity of the scale.

In the working memory task, mean accuracy decreased systematically across increasing task loads: 0-back (*M* = 0.956, *SD* = 0.024), 1-back (*M* = 0.823, *SD* = 0.045), and 2-back (*M* = 0.635, *SD* = 0.041). Correspondingly, reaction times increased with task difficulty (*RT₀* = 565.8 ± 78.8 ms; *RT₁* = 773.3 ± 76.3 ms; *RT₂* = 915.9 ± 92.3 ms). This pattern is consistent with the expected effects of increasing working memory load, indicating good discriminant validity of the task conditions. It should be noted that the 0-back, 1-back, and 2-back conditions were designed to index different levels of working memory load, rather than representing parallel items reflecting a single latent construct. Therefore, computing Cronbach’s *α* across the three conditions would be inappropriate. To evaluate the internal consistency of the task, a split-half reliability approach (odd–even trials) was applied within each condition. The resulting split-half reliability coefficients ranged from 0.78 to 0.86, indicating high measurement stability across all load levels (Redick and Lindsey, 2013). Taken together, the n-back paradigm demonstrated good internal consistency and structural validity in the present sample of older adults and can be considered a reliable measure of working memory performance. Overall, the measurement instruments showed satisfactory reliability, and task performance distributions were appropriate, meeting the statistical assumptions for subsequent correlational and moderation analyses.

### 3.3 Between-group comparisons: performance on the N-back task across physical activity levels

To examine the effects of physical activity level on working memory performance, a 3 (physical activity level: low, moderate, high) × 3 (task difficulty: 0-back, 1-back, 2-back) repeated-measures analysis of variance (ANOVA) was conducted on accuracy. Mauchly’s test of sphericity indicated a violation of the sphericity assumption (*W* = 0.839, *p* < 0.001); therefore, Greenhouse–Geisser corrections were applied. The results revealed a significant main effect of physical activity level, *F*(2, 120) = 8.83, *p* = 0.001, *η_p_^2^* = 0.128, with the high physical activity group showing significantly higher accuracy than both the low and moderate physical activity groups. A significant main effect of task difficulty was also observed, *F*(1.72, 206.72) = 2390.70, *p* < 0.001, *η_p_^2^* = 0.952, demonstrating a marked decrease in accuracy as task difficulty increased. Moreover, the interaction between physical activity level and task difficulty was significant, *F*(3.45, 206.72) = 5.07, *p* < 0.01, *η_p_^2^* = 0.078, suggesting that the effect of physical activity on working memory performance varied across different levels of task difficulty.

Simple effects analyses (see Table 4) revealed that the effect of physical activity level was significant under both the 0-back, *F*(2, 120) = 12.79, *p* < 0.001, and 2-back conditions, *F*(2, 120) = 11.82, *p* < 0.001, but not under the 1-back condition, *F*(2, 120) = 1.32, *p* = 0.271. *Post hoc* pairwise comparisons with Bonferroni correction revealed that, under the 2-back condition, the high physical activity group demonstrated significantly higher accuracy than both the low (*p* = 0.012) and moderate physical activity groups (*p* < 0.001), whereas no significant difference was observed between the low and moderate groups (*p* = 0.990). These findings indicate that the facilitating effect of physical activity on working memory performance was primarily evident under high cognitive load. These findings suggest that the facilitating effect of physical activity on working memory performance was most evident under high cognitive load. While group differences were also observed under low-load conditions, no significant differences emerged under moderate cognitive load.

**Table 4:** Pairwise comparisons of physical activity groups under the 2-back condition.

Group comparison	Mean Diff	*SE*	*p*	*η_p_^2^*	95%CI
Low vs. Moderate	0.009	0.009	0.990	0.165	[−0.013, 0.031]
Low vs. High	−**0.028**	0.010	**0.012**	**0.165**	[−0.051, −0.005]
Moderate vs. High	−**0.037**	0.008	**<0.001**	**0.165**	[−0.056, −0.018]

Mean Diff = mean difference (first group − second group). Positive values indicate higher 2-back accuracy in the first group, whereas negative values indicate higher accuracy in the second group, bold values indicate statistically significant differences (*p* < 0.05).

### 3.4 Correlation analysis

To examine the linear associations among the main variables, Pearson correlation coefficients were calculated for physical activity level (MET), social support (SSRS), and working memory performance across the 0-back, 1-back, and 2-back conditions. As shown in Table 5, physical activity level was positively correlated with social support (*r* = 0.290, *p* < 0.001). In terms of working memory performance, MET showed significant positive correlations with accuracy in both the 0-back (*r* = 0.213, *p* = 0.018) and 2-back conditions (*r* = 0.343, *p* < 0.001), whereas no significant association was observed for the 1-back condition (*r* = 0.036, *p* = 0.692). These findings suggest that physical activity is associated with working memory performance, with a stronger relationship observed under higher cognitive load. Similarly, total social support scores were significantly positively correlated with 2-back accuracy (*r* = 0.384, *p* < 0.001), but were not significantly associated with accuracy in the 1-back or 0-back conditions. In addition, exploratory analyses of the three dimensions of social support (subjective support, objective support, and support utilization) indicated similar patterns of association with working memory performance. Specifically, all dimensions showed positive associations with 2-back accuracy, consistent with the overall social support score. Overall, these findings provide support for Hypotheses H1 and H2, indicating that both physical activity level and social support are positively associated with working memory performance, particularly under higher cognitive load conditions. In addition, exploratory analyses of reaction time (RT) revealed that RT and accuracy were not consistently associated across task conditions. A significant negative correlation was observed only between RT and accuracy in the 0-back condition (*r* = −0.372, *p* < 0.001), whereas no significant associations were found in the 1-back and 2-back conditions. This pattern suggests that RT and accuracy may reflect partially distinct cognitive processes, consistent with prior literature indicating a dissociation between processing speed and accuracy in working memory tasks (Owen et al., 2005; Salthouse, 2010).

**Table 5:** Correlation matrix of main variables (*N* = 123).

Variables	1	2	3	4	5	6	7	8
1. PA (MET)	1							
2. SSRS Total Score	0.290**	1						
3. 0-back ACC	0.213*	0.142	1					
4. 1-back ACC	0.036	0.114	0.176	1				
5. 2-back ACC	0.343**	0.384**	0.188*	0.179	1			
6. 0-back RT	−0.185*	−0.106	−0.372**	−0.167	−0.100	1		
7. 1-back RT	−0.165	0.048	−0.100	−0.176	−0.032	0.196*	1	
8. 2-back RT	−0.127	−0.023	−0.062	−0.112	−0.055	−0.068	−0.054	1

*p < 0.05, **p < 0.01; *N* = 123.

**Figure 1:**
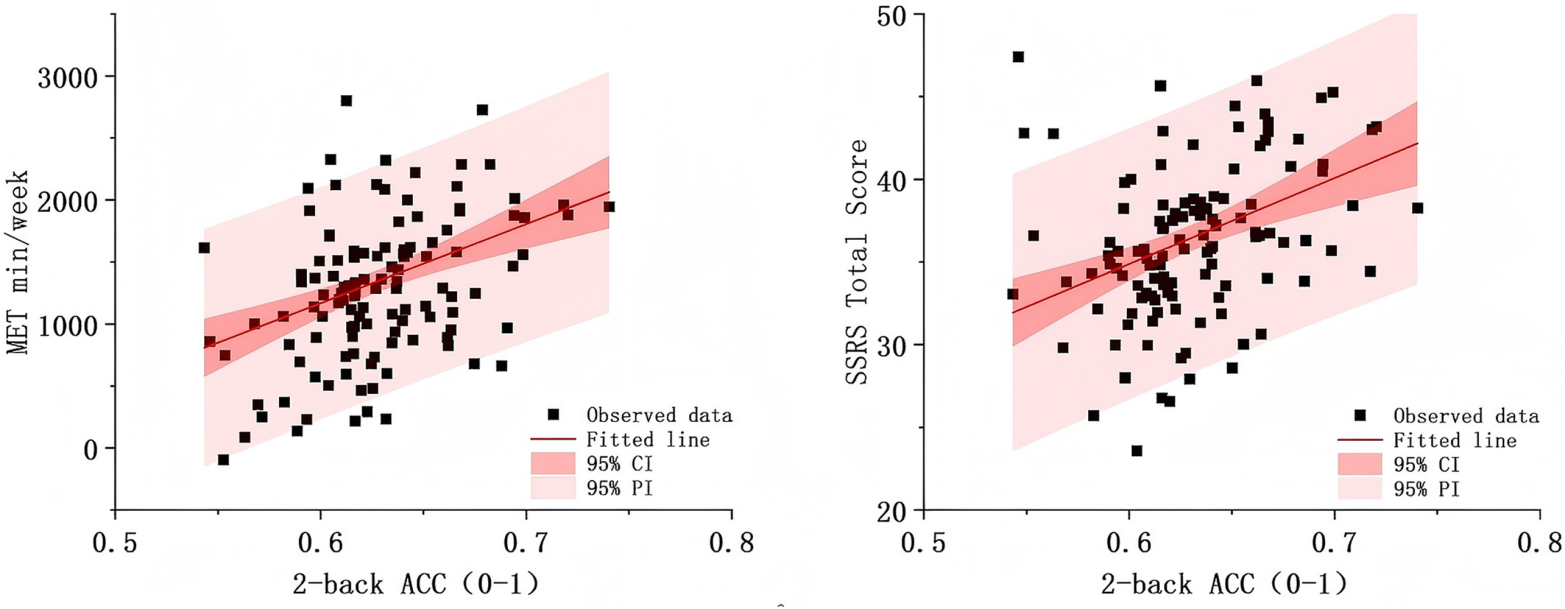
Scatterplots and linear regression lines for MET, SSRS total score, and 2-back accuracy. CI, confidence interval; PI, prediction interval.

To further provide an intuitive illustration of the linear relationships among the main variables, scatterplots with fitted regression lines were generated (see Figure 1). In both plots, the slopes of the fitted lines were positive, supporting the hypothesized positive associations of the independent variable and the moderating variable with working memory performance. The linear models satisfied assumptions of normality and residual diagnostics, indicating that the observed relationships were statistically robust.

### 3.5 Moderating effect of social support

To examine whether social support moderates the association between physical activity and working memory performance in older adults, a hierarchical multiple regression analysis was conducted using PROCESS Model 1 (version 4.1). Prior to analysis, physical activity (MET) and social support (SSRS) were z-standardized to reduce scale differences and minimize multicollinearity. Variance inflation factor (VIF) values for all predictors were below 1.3, indicating no serious multicollinearity concerns.

In the regression model, 2-back accuracy was entered as the dependent variable, physical activity level (MET) as the independent variable, and social support (SSRS) as the moderating variable, with age, sex, and years of education included as covariates. As shown in Table 6, the overall model was significant, *F*(6, 116) = 8.78, *p* < 0.001, explaining 31.23% of the variance (*R*^2^ = 0.312). physical activity (*β* = 0.217, *p* = 0.009) and social support (*β* = 0.383, *p* < 0.001) were both positively associated with 2-back accuracy. The interaction term (MET × SSRS) was significant (*β* = 0.219, *p* = 0.005), accounting for an additional 4.86% of the variance (Δ*R*^2^ = 0.0486), indicating a significant moderation effect. Bias-corrected bootstrap analyses (5,000 resamples) further confirmed the robustness of the interaction effect, with a 95% confidence interval of [0.079, 0.373], which did not include zero.

**Table 6:** Hierarchical regression and moderation analysis predicting 2-back accuracy.

Predictor	*β*	*t*	*SE*	*R^2^*/Δ*R^2^*
Step1: Control variables				*R^2^* = 0.104
Age	−0.061	−1.48	0.015	
Sex	−0.376	−2.43*	0.155	
Education level	0.154	1.59	0.097	
Step2: Main effects				Δ*R^2^* = 0.159
MET	0.217	2.640*	0.082	
SSRS	0.383	4.602***	0.083	
Step 3: Interaction effect				Δ*R^2^* = 0.049
MET×SSRS	0.219	2.864***	0.076	

*N* = 123. All tests were two-tailed. *p < 0.05, **p < 0.01, ***p < 0.001. *F*(6, 116) = 8.78, *p* < 0.001, R^2^ = 0.312.

**Figure 2:**
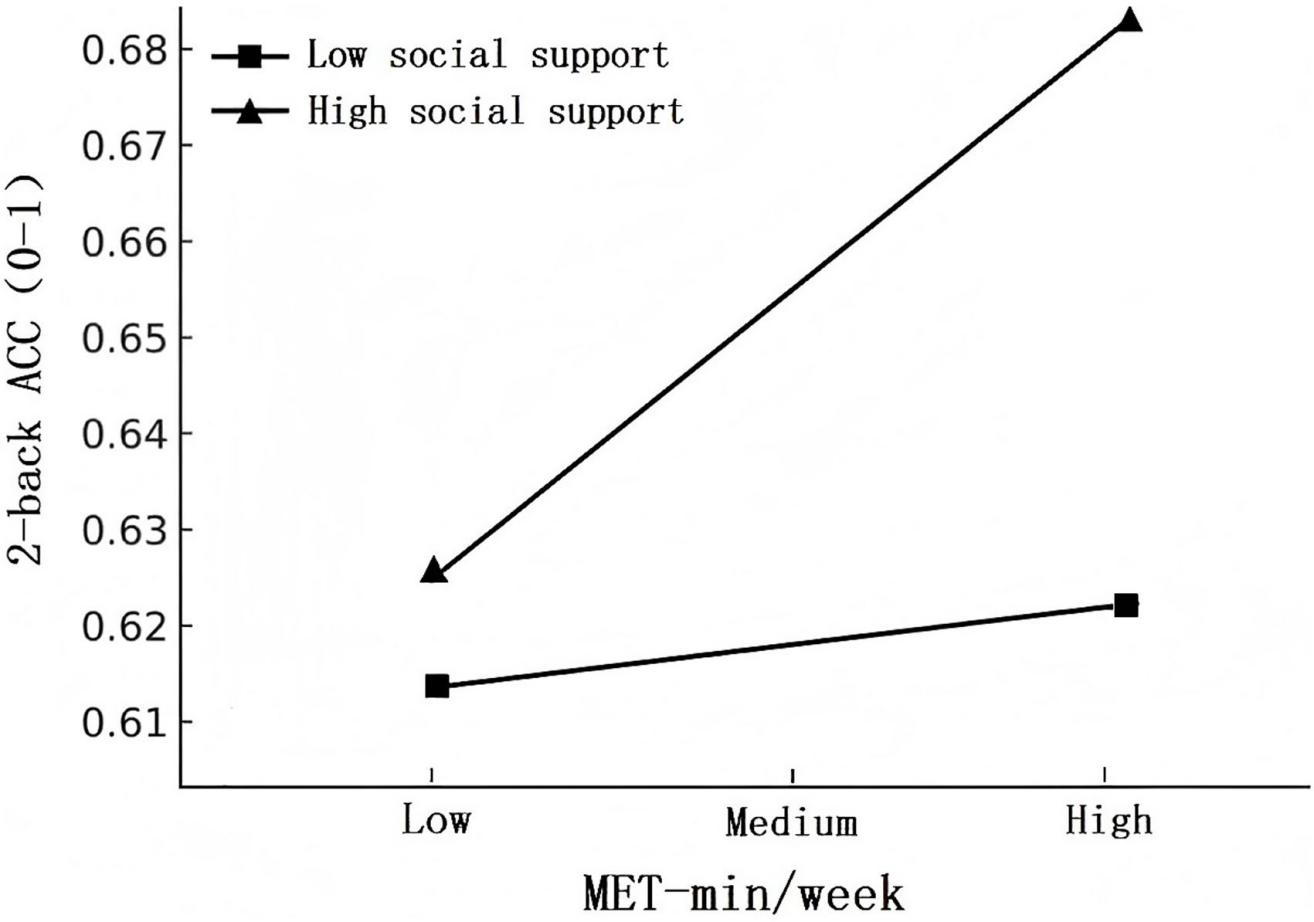
Moderating effect of social support on the association between physical activity and working memory performance.

To further clarify the direction and nature of the interaction between physical activity and social support, a simple slopes analysis was conducted following the procedure proposed by Frone et al. (1992), and the interaction effects were visualized in Figure 2. Social support was grouped based on its mean and standard deviation: participants with social support scores one standard deviation above the mean (+1 *SD*) were classified as the high social support group, those one standard deviation below the mean (−1 *SD*) as the low social support group, and those between these values as the moderate social support group.

The results indicated that physical activity was not significantly associated with working memory performance at low levels of social support (*β* = −0.002, *p* = 0.988, 95% CI [−0.238, 0.234]). However, at mean levels of social support, physical activity was significantly positively associated with 2-back accuracy (*β* = 0.217, *p* = 0.009, 95% CI [0.054, 0.380]). This association became stronger at high levels of social support (*β* = 0.436, *p* < 0.001, 95% CI [0.228, 0.643]). These findings indicate that social support significantly moderates the relationship between physical activity and working memory performance, such that the positive effect of physical activity on working memory is amplified at higher levels of social support.

## 4 Discussion

Based on a community-based sample of 123 older adults, the present study examined the associations among physical activity, social support, and working memory performance. Several key findings emerged. First, working memory accuracy declined significantly with increasing task difficulty, confirming the sensitivity sensitivity of the n-back paradigm in an older adult population. Second, physical activity level was positively associated with working memory accuracy, with participants in the high physical activity group demonstrating significantly higher accuracy than those in the low and moderate physical activity group under the 2-back condition, indicating that the cognitive benefits of physical activity were most pronounced under high cognitive load. Third, social support was positively associated with working memory performance, particularly under higher cognitive load conditions, highlighting its role as an important psychosocial correlate of cognitive functioning in later life. Finally, and most notably, social support exerted a significant moderating effect on the relationship between physical activity and working memory, such that the positive association between physical activity and cognitive performance became stronger as social support increased. As an exploratory study, the present research provides preliminary evidence regarding the associations between physical activity and social support, offering a basis for future investigation of their potential underlying mechanisms.

Taken together, these findings provide consistent support for Hypotheses H1, H2, and H3, and offer empirical evidence for the joint contributions of lifestyle and psychosocial factors to cognitive functioning in older adults. The present results extend existing research on healthy aging by demonstrating that physical activity and social support operate interact to promote working memory performance, thereby contributing to a more nuanced understanding of cognitive maintenance in later life.

### 4.1 Physical activity and working memory

The present findings indicate that higher levels of physical activity are associated with better working memory accuracy in older adults, with this facilitating effect being most pronounced under high cognitive load conditions (i.e., the 2-back task). Although some group differences were also observed under low-load conditions (0-back), these effects were not evident under moderate load (1-back). One plausible explanation for this pattern is the presence of a ceiling effect in low-demand tasks, where task difficulty may be insufficient to sensitively capture individual differences in cognitive capacity. Under higher working memory load, however, performance differences across physical activity levels became pronounced, suggesting that the cognitive benefits of physical activity are more readily expressed when task demands are high.

This pattern is consistent with cognitive load theory, which posits that the effects of individual differences and intervention-related factors become more apparent as task demands increase. Although the effect sizes of both the main effect of physical activity and its interaction with task difficulty were relatively small (*η_p_^2^* = 0.128 and *η_p_^2^* = 0.078, respectively), these effects remain theoretically and practically meaningful, particularly in aging populations. In later life, even modest cognitive advantages may accumulate over time through sustained lifestyle engagement, ultimately leading to meaningful improvements in cognitive health and functional independence. From an intervention perspective, these findings suggest that physical activity may be especially effective in enhancing performance on cognitively demanding tasks, offering valuable guidance for the design of targeted cognitive and exercise-based interventions (Colcombe and Kramer, 2003).

The observed associations align closely with a substantial body of prior research demonstrating the cognitive benefits of regular physical activity. Meta-analytic and experimental studies have shown that physical activity is associated with improvements in prefrontal and hippocampal function, thereby enhancing executive control and working memory performance (Hillman et al., 2008). These neurocognitive enhancements may to more efficient information maintenance and updating processes. Beyond these physiological mechanisms, physical activity is often accompanied by more positive affective states and greater social engagement, which may further enhance task engagement, attentional monitoring, and motivational resources, ultimately translating into higher task accuracy (Erickson et al., 2011).

In contrast to accuracy-based outcomes, the associations between physical activity and reaction time were relatively weak and limited and inconsistent only under low load significant in the present study. One possible explanation is the presence of a speed–accuracy trade-off in older adults, whereby some participants prioritize accuracy at the expense of response speed, leading to increased variability in reaction times. Additionally, age-related slowing in perceptual–motor processes, fluctuations in attentional resources, and measurement noise may further reduce the sensitivity and stability of reaction time indices relative to accuracy measures. Although the overall trend suggested faster responses among participants with higher physical activity levels, the lack of statistical significance is consistent with known characteristics of older adult samples and the psychometric properties of the n-back task, and thus represents a theoretically plausible and methodologically reasonable finding.

### 4.2 Social support and working memory

The present study found that social support was positively associated with working memory performance in older adults, particularly under higher cognitive load conditions. This suggesting that social support may serve as an important protective factor for cognitive functioning in later life. This finding is consistent with previous evidence indicating that levels of social engagement, exercise habits, and social support are significantly associated with the prevalence of cognitive impairment among older adults (Wang et al., 2023), further reinforcing the role of social support in cognitive protection.

Prior longitudinal research has demonstrated that social support is associated with slower rates of cognitive decline, even after controlling for education and physical health (Seeman et al., 2001). From a psychosocial perspective, social support may promote cognitive health through multiple pathways, including buffering stress, reducing depressive symptoms, improving sleep quality, and enhancing self-efficacy and life satisfaction (Holt-Lunstad et al., 2015). Accumulating evidence suggests that the association between social support and cognitive health is both robust across different populations and study designs (Uchino, 2006).

The correlational and regression results of the present study are broadly consistent with these previous findings. In particular, social support was significantly associated with working memory performance under higher cognitive load, and also showed a significant positive effect in the regression model. Together, these results highlight the importance of social support as a key psychosocial resource in the preservation of cognitive functioning during aging (Xue et al., 2018).

### 4.3 The moderating role of social support

The present study provides clear evidence that social support significantly moderates the relationship between physical activity and working memory performance in older adults. Results from PROCESS Model 1 indicated that the interaction term (MET × SSRS) was significant (*β* = 0.219, *p* = 0.005), with a 95% bootstrap confidence interval of [0.079, 0.373], which did not including zero. This finding indicates that higher levels of social support strengthen the positive association between physical activity and working memory performance. Simple slopes analyses further demonstrated that the positive relationship between physical activity and 2-back accuracy was significantly enhanced when social support was high, whereas this association was not significant under conditions of low social support. These results are consistent with the stress-buffering model of social support (Cohen and Wills, 1985), which posits that social support can mitigate the adverse effects of stress and amplify the benefits of health-promoting behaviors.

From a mechanistic perspective, higher levels of social support may enhance the cognitive benefits of physical activity through several complementary pathways. Social support may increase motivation for physical activity, facilitate emotional regulation, and reduce negative affect, while simultaneously providing instrumental and emotional resources that promote sustained engagement in exercise (Uchino, 2006). Individuals with greater social support are also more likely to experience higher self-efficacy, better exercise adherence, and more positive affective responses to physical activity, all of which may translate into superior working memory performance.

Overall, these findings highlight a interaction between physical activity and social support in shaping cognitive outcomes in older adults. By demonstrating that social support amplifies the cognitive benefits of physical activity, the present study contributes to a more nuanced understanding of the psychosocial mechanisms underlying cognitive health in aging, and underscores the importance of integrating social and lifestyle factors in interventions aimed at promoting healthy cognitive aging (Holt-Lunstad et al., 2015).

### 4.4 Limitations and future directions

Despite providing valuable evidence on the roles of physical activity and social support in working memory among older adults, the present study has several limitations that should be acknowledged.

First, the cross-sectional design precludes causal inferences regarding the relationships among variables. Future research could adopt longitudinal designs or randomized controlled trials to better clarify the causal pathways linking physical activity and social support to cognitive functioning, as well as to examine their dynamic changes over time.

Second, both physical activity (IPAQ-SF) and social support (SSRS) were assessed using self-report measures. Older adults may be subject to recall bias and social desirability effects when reporting behavioral frequency and duration, which may compromise data accuracy. Future studies are encouraged to incorporate objective measures (e.g., accelerometers or wearable devices) and employ multi-method approaches (i.e., combining subjective and objective assessments) to enhance ecological validity and measurement precision. In addition, differentiating types of physical activity (e.g., aerobic exercise, resistance training, and balance training) may help to clarify their potentially distinct effects on cognitive functioning.

Third, the sample was primarily drawn from urban communities and consisted of relatively well-educated participants, which may limit the generalizability of the findings. In addition, the relatively small size of the low physical activity group may have affected the stability of between-group comparisons. Future studies should aim to increase sample heterogeneity by including participants from diverse regions (e.g., urban and rural areas), educational levels, employment statuses, and socioeconomic backgrounds, as well as to optimize group balance in order to enhance the robustness and generalizability of the findings.

Fourth, although the 2-back task was used as the primary indicator of working memory and is effective in capturing moderate-to-high cognitive load, higher-load conditions (e.g., 3-back) and other cognitive domains were not included in the present study. This may have limited a more comprehensive evaluation of cognitive functioning. Future research could incorporate higher-load tasks and multidimensional cognitive measures (e.g., executive function and attentional control) to further improve sensitivity and provide a more comprehensive characterization of cognitive functioning.

Finally, the present study used total physical activity (MET) as an overall indicator and did not differentiate between types of physical activity. Future studies could further examine the differential effects of various types and intensities of physical activity (e.g., aerobic, resistance, and coordination training) on specific cognitive domains, thereby informing more targeted intervention strategies.

## 5 Conclusion

The present study demonstrates that both physical activity and social support are significant positive associated with working memory performance in older adults, with effects that are most evident under higher cognitive load (2-back condition). Moreover, social support plays a significant moderating role in the relationship between physical activity and working memory, such that the association between physical activity and cognitive performance becomes stronger as social support increases, but is not significant at low levels of social support.

## Data Availability

The raw data supporting the conclusions of this article will be made available by the authors, without undue reservation.
